# A Group Neighborhood Average Clock Synchronization Protocol for Wireless Sensor Networks

**DOI:** 10.3390/s140814744

**Published:** 2014-08-12

**Authors:** Lin Lin, Shiwei Ma, Maode Ma

**Affiliations:** 1 Department of Automation, Shanghai University, Shanghai 200072, China; E-Mail: masw@shu.edu.cn; 2 School of Electrical and Electronic Engineering, Nanyang Technological University, 639798 Singapore; E-Mail: EMDMA@ntu.edu.sg

**Keywords:** wireless sensor networks, clock synchronization, convergence, consensus clock

## Abstract

Clock synchronization is a very important issue for the applications of wireless sensor networks. The sensors need to keep a strict clock so that users can know exactly what happens in the monitoring area at the same time. This paper proposes a novel internal distributed clock synchronization solution using group neighborhood average. Each sensor node collects the offset and skew rate of the neighbors. Group averaging of offset and skew rate value are calculated instead of conventional point-to-point averaging method. The sensor node then returns compensated value back to the neighbors. The propagation delay is considered and compensated. The analytical analysis of offset and skew compensation is presented. Simulation results validate the effectiveness of the protocol and reveal that the protocol allows sensor networks to quickly establish a consensus clock and maintain a small deviation from the consensus clock.

## 1. Introduction

Wireless sensor networks (WSNs) are now widely deployed in homes, hospitals, factories, streets, battlefields, *etc.*, where they are used for a lot of monitoring applications, such as monitoring of water distribution systems [1], earthquakes [2], buildings [3], civil infrastructure [4], habitat activities [5], *etc.* In some of the applications, such as data fusion, target tracking and speed estimation, the system needs to have the knowledge of time among sensor nodes in order to determine the timing of the event. This is realized by clock synchronization (sometimes called time synchronization). Besides the application requirement, clock synchronization is important to avoid interference if the medium access control protocol uses time division medium access (TDMA) [6,7]. For the networks that utilize duty-cycling schemes and turn off the radio for energy saving, accurate time helps to save energy by shortening the necessary guard time.

Imperfections in low-cost sensor nodes will cause offsets and skew drifts. Environmental factors such as the temperature also contribute to this clock shift. Therefore, the sensors will lose synchronization with other sensor nodes. This leads to the meaninglessness of sensing data for many applications. Clock synchronization techniques are therefore needed to synchronize the sensor nodes in WSNs. The algorithms compensate the offset and skew rate by exchanging information among sensor nodes. There are several challenges to design a clock synchronization algorithm. First, since the information is shared among sensor nodes by passage exchanging, propagation delays would cause inaccuracy of time instant sharing, so a specific compensation technique is required. Second, because the sensor nodes are densely deployed in the monitoring area, the nodes can only communicate with the sink node via a multi-hop route. Designing an accurate, fast convergence and scalable global clock synchronization algorithm becomes a challenge. Third, the sensors are inexpensive devices, which may often incur failure. A dynamic and robust scheme is needed. Fourth, since sensor nodes are almost always powered by batteries, energy efficiency becomes one of the major concerns. These challenges need to be addressed in the design of any clock synchronization protocol.

In this paper, a novel distributed clock synchronization scheme, named group neighborhood averaging (GNA), is presented. It is a fully distributed, network-wide clock synchronization algorithm. Two-way communication is adopted to estimate the propagation delay and further used for offset and skew rate compensation. Without extra data exchange, just using the exchanged messages, the algorithm takes average of the offset and skew rate value among the neighboring nodes within the transmission range. The group-averaging algorithm leads to a fast convergence.

The rest of this paper is organized as follows: Section 2 discusses related work. Section 3 presents the system model and Section 4 explains the proposed GNA algorithm. Simulation results are given and discussed in Section 5. Section 6 concludes the paper and describes possible future work.

## 2. Related Work

Many dedicated strategies and protocols have been proposed to address the problem of clock synchronization in WSNs in the past decades as surveyed in [8–11]. The Network Time Protocol (NTP) [12] is one of the popular clock synchronization protocols in use. It adopts a hierarchical tree of timeservers. By recording the sending and receiving timestamps, the propagation delay is estimated. The time is then compensated using this value. NTP is not suitable for WSNs for two main reasons. First, it takes long time for the entire network to synchronize to the reference clock. Second, the accuracy is low and the complexity is relatively high. Reference Broadcast Synchronization (RBS) [13] uses a node to send a reference broadcast beacon to neighbors in the physical layer. The receivers exchange the time of arrival and compensate their own clocks. The advantage is that RBS shortens the critical path since the broadcast beacon is only used as a reference. The potential drawback lies that the exchanges of time of arrival between the neighbors incur a large overhead and the performance may decrease in large-scale multi-hop networks. In [14], by recording the sending and receiving time of the probe message, the bounds of the relative offset and drift can be obtained and then the relative offset and relative drift are estimated. It is simply a computational task. The algorithm is based on a tree topology network and the sensor nodes only synchronize with their parent fusion nodes. The disadvantage of the protocol is that it is designed only for a tree topology network, not a mesh network which is more common. Another problem is that if synchronization were required for the entire network, the convergence time would be very large. Timing-sync protocol for sensor networks (TPSN) [15] and recursive time synchronization protocol (RTSP) [16] have similar solutions. The protocols periodically elect a root node, which acts as reference clock. Root node rotation balances the energy consumption among all the sensor nodes. A tree architecture is formed for clock synchronization. A similar NTP algorithm, which uses four time stamps to estimate the propagation delay, is adopted. The disadvantage is that the level discovery phase consumes quite a large amount of energy and time, which leads to slow convergence. In addition, due to the hierarchical topology, the protocol is prone to node failure. The flooding time synchronization protocol (FTSP) [17] also randomly elects a root node, but for time synchronization, either single hop or multi-hop, FTSP adopts one way synchronization. The message delivery delay is decomposed into interrupt handling of CPU, encoding and decoding of radio chip to transform the message, byte alignment of radio chip at the receiver side and the propagation delay. The time stamping effectively reduces the error introduced by interrupt handing, encoding and decoding. Compared to them, propagation is relatively small and neglected. Time-diffusion synchronization protocol (TDP) [18] uses NTP-like information exchange and spreads the reference time to the whole network. The performance is good, but the algorithm is complicated and not energy efficient. In summary, for tree hierarchical clock synchronizations, the disadvantages include slow convergence, energy waste and complexity of root node election, proneness to node and link failure and difficulties for mobile scenarios.

Distributed consensus-based protocols for global clock synchronization have been proposed in [19–23]. In [21], local synchronization is conducted by averaging the offset and finally the entire network converges to a virtual consensus clock, but only clock offset is compensated. Clock drift is not discussed. References [19] and [22] consider both offset and clock drift compensation. Each node periodically broadcasts a synchronization beacon. The receiving node takes an average of the offset and drift. The solution is fully distributed without the need of a root node. All nodes run the same algorithm. The protocol is robust to dynamic *ad-hoc* networks issues such as node and link failure. In [20], the algorithm is further improved. A confidence weight parameter is introduced for the average calculation. The node, which has conducted an averaging calculation, would be given a big weight. In [23], the protocol adopts max consensus to compensate for clock drift but the reason is not clearly presented. One limitation in the works presented in [19–22] is that the protocol is designed in the absence of propagation delay. However, the propagation delay is a very important issue, which cannot be ignored in real clock synchronization scenarios. Another limitation is that they take averaging only between one sender and one receiver at each time. This leads to a slow convergence. The authors of [24] use a group average and assume the propagation delay follows Gaussian distribution. However, the algorithm only achieves good performance when the network is time delay balanced. The reason is that the averaging operation for a Gaussian distributed delay approaches zero. This method would not be suitable for an unbalanced network. [25] considers both fixed and random propagation delays and proposes a distributed synchronization algorithm. By using the maximum likelihood estimation and belief propagation on graphical models, global clock synchronization is achieved. [26] improves it and proposes an asynchronous algorithm. Different nodes could update their estimates based on different frequencies. For these two methods, the algorithms are relatively complicated and need more storage and computational resources.

This paper proposes a fully distributed, network-wide clock synchronization scheme. The scheme is scalable and not constrained to a certain network topology. It is also robust to node failure. Two-way communication is adopted to estimate the propagation delay. The exchanged messages are multiply used for offset and skew rate compensation. Nodes take turns to ask their neighbors for clock information. Then they average all the received offset and skew rate values in each round and broadcast the values back to their neighbors. A fast convergence is achieved by the proposed group-averaging algorithm. The procedure of the algorithm is clearly presented. It has the advantages of accuracy, fast convergence, low complexity and easy implementation. The main contributions of this paper are:

(1) Group averaging is used instead of point-to-point averaging for faster convergence.
(2) Propagation delay is compensated compared with several important related papers.
(3) Minimum data exchange is achieved for both offset and skew rate compensation, and at the same time the propagation delay is considered and compensated.

## 3. System Model

**Figure 1.**
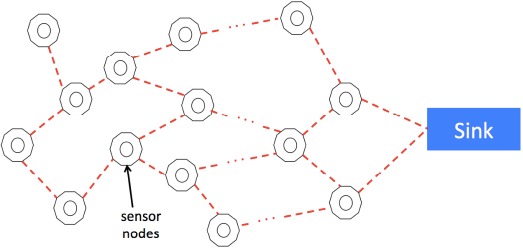
System model.

A typical WSN is composed of tens, hundreds or even up to thousands of sensor nodes which are deployed in an *ad-hoc* fashion without careful planning, as shown in Figure 1. The sensor nodes collect and transmit the sensing data to a sink node. The sink node stores and processes the data. As discussed in Section 1, the sensor nodes need to store the time instants of the data collection so that later the sink node can aggregate and process the data from different sensor nodes. Therefore, the requirement that all the sensor nodes strictly keep a common clock becomes essential.

For each sensor node, we define the clock model as Equation (1). *C*(*t*) is the clock reading at time *t*. α is the skew rate and β is the offset. In the ideal situation, α is equal to 1 and β is equal to 0:

(1) C(t)=α*t+β

The clocks in the sensor network may not be consistent for several reasons. First, the clock may drift, which can be caused by the manufacture of the oscillator components. Second, the clock could change due to some environmental condition such as temperature, pressure, battery voltage, *etc.* Third, the clocks from different sensor nodes may not be synchronized well at the beginning of the network deployment. All these reasons lead to clock readings varying from the real value. The clock reading *C_i_*(*t*) of node *i* is then expressed in Equation (2):

(2) $$C_{i}\left(t\right)=\alpha_{i}*t+\beta_{i}$$

The aim is to find the compensated α̂_*i*_ and β̂_*i*_, so as to make the compensated clock (*Ĉ_i_*(*t*) expressed in Equation (3)) to converge to a consensus clock *C_c_*(*t*) (as shown in Equation (4)):

(3) $${Ĉ}_{i}\left(t\right)=\frac{\alpha_{i}}{{\hat{\alpha }}_{i}}*t+\beta_{i}-{\hat{\beta }}_{i}$$

(4) $$\underset{t\to \infty }{\lim}{Ĉ}_{i}\left(t\right)=C_{c}\left(t\right)$$

Consensus clock is a virtual clock within the sensor network. For most WSN applications, there is no need for the clocks of sensor nodes to converge to the real clock. Take speed estimation as an example. As long as all sensor nodes are synchronized, they can successfully locate the target, and therefore successfully estimate the moving speed. In this case clock synchronization to a virtual clock is enough.

**Figure 2.**
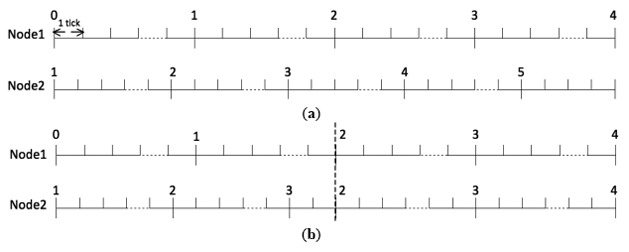
An example of offset and skew rate compensation. (**a**) Before clock synchronization; (**b**) After clock synchronization.

Figure 2 shows an example of offset and skew rate compensation. In Figure 2a, before clock synchronization, Node 1 and Node 2 have different offset and skew rates. For offset compensation, clock readings “2” for Node 1 and Node 2 are not aligned. For skew rate, it is equal to the multiplication of tick duration, $\;T_{tick}^{i}$, and counter limit, *N_i_*, as shown in Equation (5). Either different $T_{tick}^{i}$ or different *N_i_* could make the two nodes have different α*_i_*:

(5) $$\alpha_{i}=N_{i}\times T_{tick}^{i}$$

After clock synchronization, as shown in Figure 2b, Node 2 is synchronized to Node 1. For offset compensation, the two nodes align the start time of the clock reading “2”. For skew rate compensation, Node 2 adjusts its counter limit to make the clock duration equal to that of Node 1. It should be noted that only the counter limit can be adjusted by users. Tick duration is a natural property of the oscillator and therefore cannot be modified.

## 4. The Proposed GNA Algorithm for Clock Synchronization

The GNA algorithm includes two parts: offset compensation and skew rate compensation. They are realized by clock exchange and computation. The clocks of the sensor nodes will eventually converge to a consensus clock. One thing that should be noted for clock exchange among sensor nodes, is that the propagation delay incurs errors and this leads to inaccuracy. This will be compensated by two-way message exchange.

### 4.1. Propagation Delay Model

**Figure 3.**
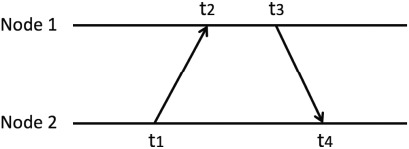
Two-way message exchange.

The signal transmission from one node to another experiences a propagation delay. This delay would cause the inaccuracy for clock synchronization between two nodes [15–17]. The idea for compensating this propagation delay is using two-way message exchange. As shown in Figure 3, Node 2 asks Node 1 for time. Node 2 records the time instant *t_1_*, when it sends message. Node 1 receives the message and records the instant *t_2_*. Then Node 1 returns the time information message and records the time instant *t_3_*. Node 2 receives it and keeps the time instant *t_4_*. Using these four time instants, Node 2 would estimate the propagation delay using Equation (6). Then it can compensate for the time offset. In the remainder of this paper, all the message exchanges adopt two-way message exchange. This is minimum information exchange for propagation delay estimation.

(6) $$\text{delay}=\;\frac{\left(t4-t1\right)-\left(t3-t2\right)}{2}$$

### 4.2. Offset Compensation

Offset compensation is to make the sensor nodes to align at a common value of time. It is equivalent to obtaining β̂_*i*_ to satisfy Equation (4). We expand it as illustrated in Equation (7):

(7) $$\begin{array}{ll} \underset{t\to \infty }{\lim}\frac{\alpha_{i}}{{\hat{\alpha }}_{i}}*t+\beta_{i}-{\hat{\beta }}_{i} & =C_{c}\left(t\right)\\ & =\alpha_{c}*t+\beta_{c} \end{array}$$

We have:

(8) $${\hat{\beta }}_{i}=\beta_{i}-\beta_{c}$$

Since sensor networks normally have a large number of sensor nodes covering a relatively big area, is impossible for a certain node to directly communicate with all the nodes in a single hop way. Therefore, local information exchange and averaging is used in this algorithm. The nodes take turns to collect time information from their neighbors and take an average of the collected time information and their own value. The result is estimated as the consensus value of the node and neighbors. The algorithm of offset compensation is presented as below:

Step 1 Peyriodically sense the channel, if the channel is clear, then broadcast a requesting packet to ask the neighbors within its transmission range for the clock information.
Step 2 The neighbors return the clock values.
Step 3 Compensate for the propagation delay
Step 4 The node calculates the average offset of all the neighbors as well as itself.
Step 5 Update its own offset value.
Step 6 Broadcast the compensation table. The table includes node ID and compensated offset.
Step 7 The neighbors receive the table and update their own clock offset.
Step 8 Repeat.

**Figure 4.**
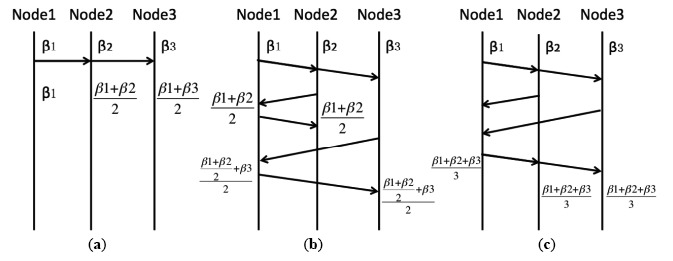
Explanation of GNA algorithm. (**a**) 2-node synchronization without considering propagation delay; (**b**) 2-node synchronization with propagation delay compensation; (**c**) group neighborhood averaging with propagation delay compensation.

In this paper, 2-node offset averaging which is used in [19–22] is replaced by group neighborhood averaging with propagation delay compensation. As shown in Figure 4, it is assumed that Node 1 is the randomly selected node which synchronizes neighboring nodes. In Figure 4a, 2-node offset averaging is realized by broadcasting the synchronization message. Node 2 and Node 3 receive this message and calculate the average of the offsets of Node 1 and themselves. In this model, the propagation delay is ignored. The three nodes obtain different clock values and the deviation of errors becomes smaller. This method is used in [19–22]. In Figure 4b, two-way message exchange is adopted to compensate for the propagation delay between two nodes. Node 1 broadcasts a request message and records the time instant. Assume Node 2 replies earlier. Node 1 takes the average of Node 1 and Node 2's offsets. Then Node 1 sends the clock and propagation delay information to Node 2. Node 2 is synchronized with Node 1. Next Node 3 follows the same procedure. In this scenario, Node 1 needs to send different synchronization messages to every neighboring node, and these nodes do not converge to a single clock. In Figure 4c which is proposed by this paper, Node 1 waits to collect all the clock information from neighboring nodes. It calculates the propagation delay and takes the group average among all the nodes. In this way, an accurate, fast offset convergence is achieved. It should be mentioned that because there are three time message exchanges between the randomly elected node and its neighbor, one can use the first two message exchanges to estimate and compensate the propagation delay. There is no extra information exchange needed, therefore minimum information exchange is adopted for both propagation delay compensation and offset compensation.

Next, it will be shown that the algorithm of offset compensation achieves global synchronization throughout the entire network. As defined in Section 2, β*_i_* is the offset value of node *i*. It can be obtained as below:

#### Theorem 1

The offset values of all the sensor nodes in the network converge to certain value.

#### Proof

Assume that $\beta_{max}^{0}$ and $\beta_{min}^{0}$ are the maximum and minimum offset values among all the sensor nodes in the network at the initial time. $\beta_{max}^{n}$ and $\beta_{min}^{n}$ are the maximum value and minimum value among the offset values of all the sensor nodes after the *n*th round synchronization. For the (*n* + 1)th round of synchronization, each node conducts the group averaging within its transmission range. The maximum offset value would be averaged with some other offset values which are smaller than or equal to it. Then the result should be smaller than or equal to $\beta_{max}^{n}$. If this result is the new maximum offset value after the (*n* + 1)th round of synchronization, we can say $\beta_{max}^{n+1}\leq \beta_{max}^{n}$. If $\beta_{max}^{n+1}$ is from the averaging operation group without $\beta_{max}^{n}$ node, since the offset values in the group is smaller than or equal to $\beta_{max}^{n}$, we can also obtain $\beta_{max}^{n+1}\leq \beta_{max}^{n}$. $\beta_{max}^{n+1}$ is equal to $\beta_{max}^{n}$ only when all the offset values of the neighbors are equal to $\beta_{max}^{n}$. From $\beta_{max}^{0}$, to $\beta_{max}^{n}$, until $\beta_{max}^{+\infty }$, the maximum offset value is non-increasing over the whole synchronization time. Similarly, we have $\beta_{min}^{n+1}\geq \beta_{min}^{n}$, meaning that from $\beta_{min}^{0}$, to $\beta_{min}^{n}$, until $\beta_{min}^{+\infty }$, the minimum offset value is non-decreasing over the whole synchronization time. Because the averaging operation is conducted all the time, we can obtain that $\lim_{n\to +\infty }\beta_{max}^{n}=\lim_{n\to +\infty }\beta_{min}^{n}=\beta_{c}$. This demonstrates that the offset values of all the sensor nodes converge to the certain value β*_c_* finally.

Next we discuss the relationship between β*_c_* and the initial average offset value of all the sensor nodes $\beta_{avg}^{0}$. For each neighborhood averaging, the average value of this sensor group does not change. Similarly, for each round of synchronization, the average value of the entire network does not change. Then we have $\beta_{avg}^{0}=\beta_{avg}^{n}=\beta_{avg}^{\infty }$. As discussed above, after certain rounds of synchronization, the offset values of all the sensors converge to β*_c_*. Then the average offset value of these sensors would be $\frac{1}{k}{\text{∑}}_{i=1}^{k}\beta_{c}=\beta_{c}$. Therefore, we obtain $\beta_{c}=\beta_{avg}^{\infty }=\beta_{avg}^{0}$. The offset values of all the sensor nodes in the network converge to the initial average offset value.

MAC layer time stamping is adopted in the proposed protocol. It is well explained in detail in [19,22]. On the transmitter side, the timestamp is recorded into the message just before the packet is transmitted into the air at the physical layer. On the receiver side, once the message “Start Frame Delimiter” (SFD) is received, the instant time is recorded as the start of the packet reception. This mechanism significantly reduces the unpredictable delays in the protocol stacks at both transmitter and receiver sides. The computational complexity of the algorithm T(n) = 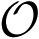 (n), where n is the number of nodes within the transmission range of a sensor node.

### 4.3. Skew Rate Compensation

Offset compensation is for time alignment among the network nodes. The skew rate also needs to be synchronized so that the sensor nodes can have a common clock speed. The method is the same as offset compensation by taking average of the skew rate values among the sensor nodes as illustrated in Equation (9):

(9) $${\hat{\alpha }}_{i}=\frac{1}{N}\sum_{i=1}^{N}\alpha_{i}$$

The algorithm of skew rate compensation is given below:

Step 1 Periodically sense the channel, if channel is clear, then broadcast a requesting packet to ask the neighbors within its transmission range for the skew rate.
Step 2 The neighbors return the skew rate.
Step 3 Compensate for the propagation delay
Step 4 The node calculates the average skew rate for all these nodes of the neighbors and itself.
Step 5 Update its skew rate value.
Step 6 Broadcast the compensation table. The table includes node ID and compensated skew rate.
Step 7 The neighbors receive the table and update their own skew rates.
Step 8 Repeat.

The skew rates of sensor nodes in the network will converge finally to a certain value. The proof is the same as Theorem 1. The computational complexity of the algorithm *T*(*n*) = 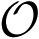 (*n*), where n is the number of nodes within the transmission range of a sensor node. This is the same as the reference protocols, FWA and CWA.

**Figure 5.**
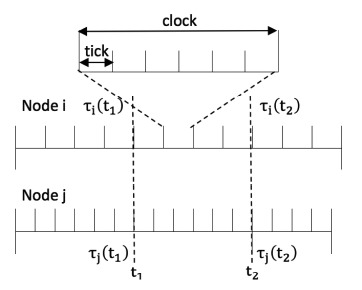
An example of skew rate compensation.

To illustrate the skew rate compensation clearly, we take an example of two-node skew rate compensation. We collect the clock readings *τ_i_*(*t*_1_) and *τ_j_*(*t*_1_) at the absolute time *t*_1_ from node *i* and node *j*, and the clock readings *τ_i_*(*t*_2_) and *τ_j_*(*t*_2_) at the absolute time *t*_2_ as shown in Figure 5 (clock readings collected at the same time are achieved by delay estimation mentioned in Part A in this section. The algorithm demonstrates that there is a two-way message exchange). *N_i_* and *N_j_* are the maximum limit of the clocks of node *i* and node *j*. When the counter reaches the maximum limit, an interrupt is triggered and the clock value increments. The relationship of the clock duration *T_clock_*, tick duration *T_tick_* and counter limit *N* is given in Equation (10).

(10) $$T_{\text{clock}}=N\times T_{\text{tick}}$$

For skew rate synchronization, based on the pre-defined counter limits *N_i_, N_j_* and the collected clock readings τ*_i_*(*t*_1_), τ*_j_*(*t*_1_), τ*_i_*(*t*_2_) and τ*_j_*(*t*_2_), the goal is to find $N_{j}^{+}$, which is the compensated counter limit of node *j*, to satisfy $T_{\text{clock}}^{i}={T_{\text{clock}}^{j}}^{+}$.

For node *i*, the duration between the two probe messages, *t*_2_ − *t*_1_, can be expressed as Equation (11). Similarly, *t*_2_ − *t*_1_ can also be expressed from node *j* as shown in Equation (12):

(11) $$t_{2}-t_{1}=\left(\tau_{i}\left(t_{2}\right)-\tau_{i}\left(t_{1}\right)\right)\times N_{i}\times T_{\text{tick}}^{i}$$

(12) $$t_{2}-t_{1}=\left(\tau_{j}\left(t_{2}\right)-\tau_{j}\left(t_{1}\right)\right)\times N_{j}\times T_{\text{tick}}^{j}$$

Combining Equations (11) and (12), we have:

(13) $$\frac{\tau_{i}\left(t_{2}\right)-\tau_{i}\left(t_{1}\right)}{\tau_{j}\left(t_{2}\right)-\tau_{j}\left(t_{1}\right)}=\frac{N_{j}\times T_{\text{tick}}^{j}}{N_{i}\times T_{\text{tick}}^{i}}=\frac{T_{\text{tick}}^{j}}{N_{i}\times T_{\text{tick}}^{i}}\times N_{j}$$

Since the goal is to satisfy $T_{\text{clock}}^{i}={T_{\text{clock}}^{j}}^{+}$, we expand it as Equation (14):

(14) $$N_{i}\times T_{\text{tick}}^{i}=N_{j}^{+}\times T_{\text{tick}}^{j}$$

Combining Equations (13) and (14), the new counter limit of node *j*'s oscillator is obtained in Equation (15):

(15) $$N_{j}^{+}=\frac{\tau_{j}\left(t_{2}\right)-\tau_{j}\left(t_{1}\right)}{\tau_{i}\left(t_{2}\right)-\tau_{i}\left(t_{1}\right)}\times N_{j}$$

The above discussion is to synchronize node *j* to node *i*. If the average compensation is desired, then the goal becomes to find $N_{i}^{+}$ and $N_{j}^{+}$, which satisfies Equation (16):

(16) $${T_{\text{clock}}^{i}}^{+}={T_{\text{clock}}^{j}}^{+}=\frac{T_{\text{clock}}^{i}+T_{\text{clock}}^{j}}{2}$$

Since *T_clock_* = *N* × *T_tick_*, for node *i* we have:

(17) $$T_{\text{tick}}^{i}\times {N_{i}}^{+}=\frac{N_{i}\times T_{\text{tick}}^{i}+N_{j}\times T_{\text{tick}}^{j}}{2}$$

By dividing $T_{\text{tick}}^{i}$ on both sides of Equation (17) and combining it with Equation (13), we obtain:

(18) $${N_{i}}^{+}=\frac{1+\frac{\tau_{i}\left(t_{2}\right)-\tau_{i}\left(t_{1}\right)}{\tau_{j}\left(t_{2}\right)-\tau_{j}\left(t_{1}\right)}}{2}\times N_{i}$$

Similarly:

(19) $${N_{j}}^{+}=\frac{1+\frac{\tau_{j}\left(t_{2}\right)-\tau_{j}\left(t_{1}\right)}{\tau_{i}\left(t_{2}\right)-\tau_{i}\left(t_{1}\right)}}{2}\times N_{j}$$

So far, the skew rates are compensated to a consensus value for the two nodes by adjusting the counter limit. This method can be extended to more nodes. After a certain number of compensation rounds, the entire network would eventually be synchronized. It should be noted that for neighboring information exchange, it is a two-way communication. By using the recorded clock values, the propagation delay can be estimated. Using the estimated propagation delay value, the clock readings of different nodes at the same time, e.g., τ*_i_*(*t*_1_) and τ*_j_*(*t*_1_), can be obtained. In Section 4 the algorithms are evaluated by simulations and the results are analyzed.

## 5. Simulation Evaluation

The algorithm of GNA clock synchronization has been simulated by MATLAB and compared with forward weighted average (FWA) and confidence weighted average (CWA) which are proposed in [20]. For FWA, Each node periodically broadcasts a synchronization beacon. The receiving node takes average of offset and drift. FWA calculates the average only between two nodes, as expressed as Equation (20). Ĉ_i_(t) is the estimation of C_i_(t). C_i_(t) and C_j_(t) are the clock readings from node *i* and node *j*. CWA is the improved algorithm based on FWA. CWA includes a confidence parameter which gives more weighting to the nodes that conduct more rounds of synchronization. It is expressed in Equation (21). The initial values of γ_i_ and γ_j_ are set to 1:

(20) $${Ĉ}_{i}\left(t\right)=\frac{C_{i}\left(t\right)+C_{j}\left(t\right)}{2}$$

(21) $$\gamma_{i}=\gamma_{i}+1$$

A 10 × 10 grid sensor network is built up. After two minutes, the synchronization procedure starts and it performs the algorithm every minute for ten rounds. The simulation parameters are described in Table 1. Several cases are discussed to evaluate the proposed GNA algorithm.

### Case 1: α*_i_* = 1, β*_i_* ≠ 0

**Figure 6.**
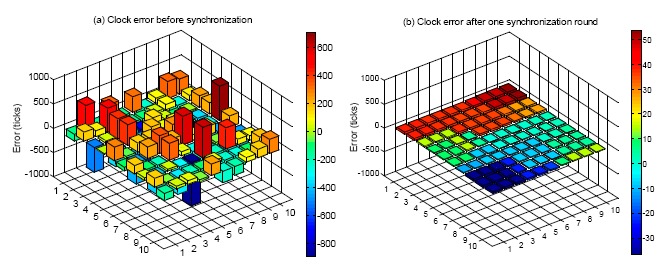
Geographic distribution of synchronization error before and after the first round of offset compensation.

In Case 1, α*_i_* is equal to 1, while β*_i_* is not equal to 0. The skew rates of all the nodes in the network are always the same. An initial error following normal distribution is given to all the sensor nodes. Figure 6 shows the geographic distribution of synchronization error before and after the first round of offset compensation. It can be seen that before the offset synchronization, the range of clock error is from −600 to +600 ticks. After one round of synchronization, the range of clock error reduces to −20 to +40 ticks. The offset is significantly compensated. It is also noted that after the offset synchronization, the values are the same for several node clusters. This is because the average algorithm is conducted for all the nodes within the transmission range of a central node. Then after the average calculation for one cluster, the nodes within the cluster would have a common clock.

**Figure 7.**
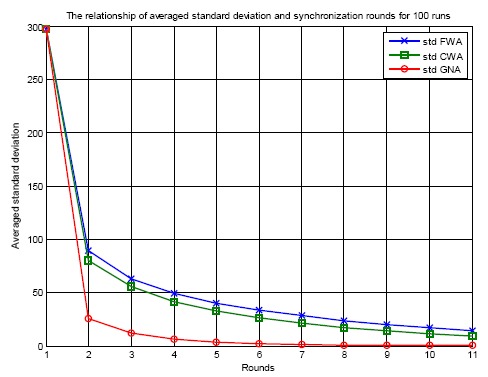
Averaged standard deviation of FWA, CWA and GNA for 10 rounds of clock synchronization.

Figure 7 shows the relationship of the averaged standard deviation of errors and the synchronization rounds for 100 runs for three clock synchronization algorithms: FWA, CWA and GNA. For each run, random initial errors are generated for each sensor node. The standard deviation is calculated as in Equation (22):

(22) $$std=\sqrt{\frac{1}{n-1}\sum_{i=1}^{n}{\left(x_{i}-\bar{x}\right)}^{2}}$$

where: $\bar{x}=\frac{1}{n}\sum_{i=1}^{n}x_{i}$.

After each round of synchronization, the standard deviation of clock offsets for a specific clock synchronization protocol is calculated and plotted. The standard deviation expresses the convergence performance by calculating how much variation exists from the average. A low standard deviation indicates that the errors of the sensor nodes tend to be very close to the mean, while a high standard deviation indicates that the errors are spread out over a large range of values. From Figure 7 it can be seen that the averaged standard deviation of the errors in the beginning is 290 ticks. It decreases as the synchronization is conducted round by round. As the synchronization is conducted, the standard deviation reduction trend becomes slow and the value goes towards zero. This is realized by the averaging behavior for the three clock synchronization algorithms. The algorithms average the clock offsets between two nodes or among a number of sensor nodes, therefore the standard deviation reduces. The performance of the proposed GNA protocol is better than that of FWA and CWA. For each round of clock synchronization, GNA achieves a lower standard deviation than FWA and CWA. This is because the offset averaging calculation is conducted among all the neighbor nodes and the obtained value is assigned to these sensor nodes when using GNA algorithm, but for FWA and CWA the average is taken only between a pair of nodes for each round. The speed of convergence of FWA and CWA is slower than that of GNA.

**Figure 8.**
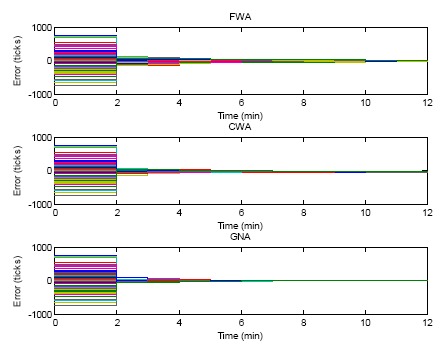
Errors of FWA, CWA and GNA with only offset compensation but without skew rate compensation.

The case of the errors in ticks of the FWA, CWA and GNA algorithm for 10 rounds of synchronization are shown in Figure 8. For each round of clock synchronization, the offsets are compensated. Because the skew rates in this case are set to 1, the tick errors would last until the next clock synchronization. The GNA algorithm achieves the fastest convergence among the three algorithms. The error converges to zero after two rounds of clock synchronization (3 min after the simulation). Zero means that the sensor nodes converge to a common virtual clock. FWA and CWA need a longer time to converge. From the figure it can be seen that FWA and CWA do not converge to zero even after 10 rounds of clock synchronization. As discussed above, the better performance of GNA is because GNA takes average of all neighbor nodes but FWA and CWA only calculate the average between two nodes.

### Case 2: α*_i_* ≠ 1, β*_i_* = 0

**Figure 9.**
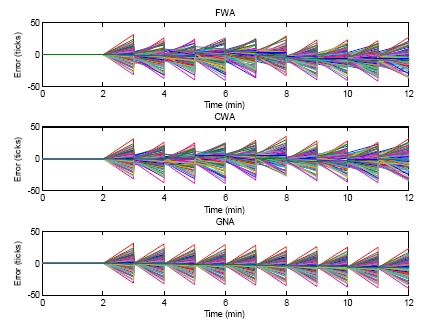
Errors of FWA, CWA and GNA with only offset compensation but without skew rate compensation.

In Case 2, there is no initial offset given to the sensor nodes in the network. Therefore, β*_i_* is equal to 0, but α, the skew rate of the sensor nodes clock is assigned a random value, which is not equal to 1, for each sensor node. The off-center skew rate causes clock errors. The clock errors for FWA, CWA and GNA algorithms within 12 min are compared in Figure 9. In this figure, only offsets are compensated, while the skew rates are not compensated. From the figure there are no initial errors in the first two minutes of the simulation. After that a random skew rate is generated for all the sensor nodes. Due to this skew rate, the errors increase in the next minute. At the third minute of the simulation, the three algorithms conduct the offset compensation. The clock errors of the sensor nodes are reduced, but because there is no skew rate compensation, the clock errors increase again in the next minute. The clock error patterns come out periodically. From the figure, for each clock synchronization at the integral time instant, the proposed GNA outperforms than FWA and CWA by obtaining a smaller clock error deviation of all the sensor nodes for each round of synchronization. As mentioned above, group averaging is the reason for GNA's better performance.

**Figure 10.**
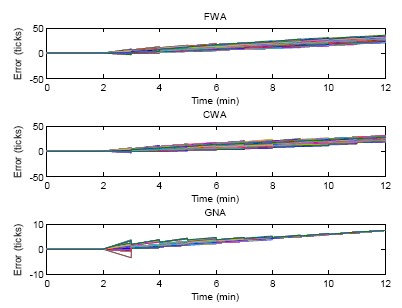
Errors of FWA, CWA and GNA of both offset compensation and skew rate compensation.

Figure 10 gives the simulation result of the comparisons of FWA, CWA and GNA for both offset and skew rate compensation. At the time instant of 2 min random skew rates are initialized for all the sensor nodes. The clock errors increase in the next minute. At the time instant of 3 min, The FWA, CWA and GNA algorithms are executed for both offset compensation and skew rate compensation. The clock errors therefore become smaller. Since one round of skew rate compensation cannot make all sensor nodes achieve a common skew rate, the clock errors still deviate in the following minutes. Then the next round of synchronization comes. The offsets and skew rates of sensor nodes are compensated again. From the figure it can be seen that at the end of the simulation, the clock errors of all the three algorithms are synchronized to a virtual consensus clock which is already not the real time.

This is the nature of the consensus algorithm. The consensus algorithm can make the clocks of sensor nodes converge, but cannot guarantee that they will converge to the real time since there is no reference clock in the network. However, this is not an issue for most applications which require only internal clock synchronization. The convergence performance of GNA is much better than that of FWA and CWA. The two-node synchronization cannot make the clock converge.

**Figure 11.**
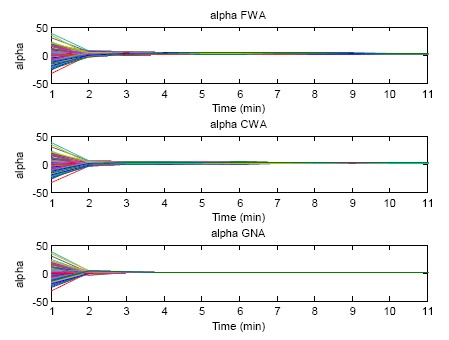
The skew rate of FWA, CWA and NA for the 10 round clock synchronizations.

In Figure 11, the skew rates of the proposed GNA, FWA and CWA are compared. Initial skew rates are generated at the beginning of the simulation. After 10 rounds of synchronization, the deviation of the skew rate becomes smaller. The proposed GNA converges faster than FWA and CWA. After six rounds of synchronization, the skew rate of GNA converges to a common value, but for FWA and CWA, the skew rates do not converge to a common value even after 10 rounds of synchronization. Group averaging within the transmission range is still the reason for the fast convergence of GNA.

### Case 3: α*_i_* ≠ 1, β*_i_* ≠ 0

**Figure 12.**
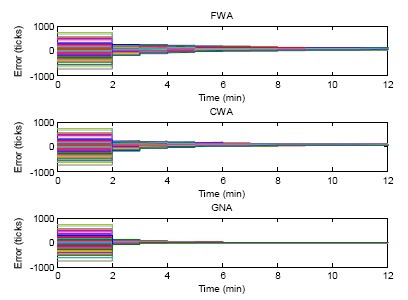
Errors of FWA, CWA and GNA of both offset compensation and skew rate compensation with initial offset and skew rate errors.

In Case 3, both initial errors and skew rates are assigned to all the sensor nodes. The errors in ticks of FWA, CWA and GNA algorithm for 10 round synchronizations are shown in Figure 12. In this simulation both offset and skew rate compensation are conducted for FWA, CWA and GNA. The initial clock errors are assigned to the sensor nodes in the beginning. After two minutes, the first round of offset and skew rate compensation are conducted. The clock errors decrease. During the next minute, due to the existence of skew rate deviation, the clock errors increase. In this figure this clock error increase cannot be clearly seen because the increase is not that significant compared with the initial errors with the range of −1000 to 1000 ticks. After 10 rounds of offset and skew rate compensation, the clocks of the sensor nodes in the network converge. It can be seen that GNA converges faster than FWA and CWA.

**Figure 13.**
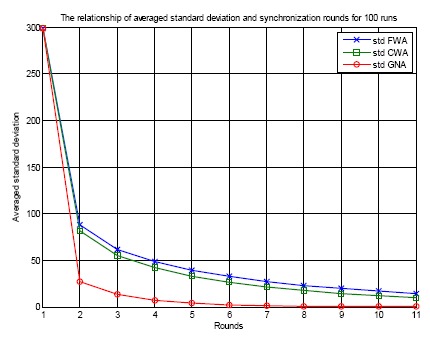
Averaged standard deviation of FWA, CWA and GNA for 10 rounds of synchronization.

Figure 13 shows the averaged standard deviation of clock errors for FWA, CWA and the proposed GNA algorithm during the 10 rounds of offset and skew rate compensation for 100 runs. For each run random initial errors are generated for all the sensor nodes. This result indicates a similar explanation as in Figure 7. At the beginning of the simulation, the standard deviation of the clock errors of the sensor nodes are big, here around 300 ticks. After one round of synchronization, the standard deviation drops significantly to around 50. As time collapses, the standard deviation continues to decrease towards zero. This illustrates that the algorithms of FWA, CWA and the proposed GNA are effective for the purpose of clock synchronization for WSNs. From the figure it can also be seen that the proposed GNA achieves better performance of convergence than FWA and CWA.

**Figure 14.**
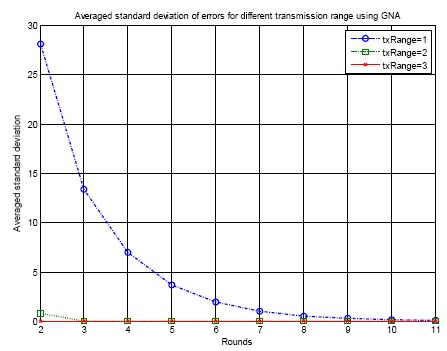
Averaged standard deviation of errors for different transmission range using GNA.

Figure 14 shows the averaged standard deviation of errors for different transmission ranges using the proposed GNA for 100 runs. Transmission ranges equal to 1, 2 and 3 unit length are simulated. From the figure when the transmission range is equal to 1, the averaged standard deviation in the beginning is 28 ticks. As time collapses, the standard deviation decreases significantly. After ten rounds of synchronization, the standard deviation drops to zero. When the transmission range increases to 2 or 3, the performance of convergence becomes significantly better. This is because when the transmission range increases, more nodes are involved for the average calculation. *N* in Equations (6) and (7) becomes larger. The convergence becomes faster throughout the entire network.

**Figure 15.**
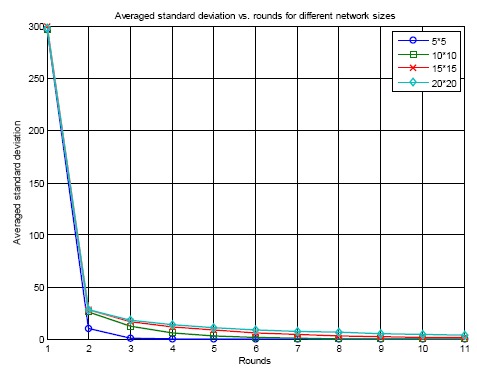
Averaged standard deviation of errors for different network sizes using GNA.

Figure 15 shows the averaged standard deviation of errors *vs.* number of rounds for different network sizes using the GNA algorithm. There are four scenarios: 5 × 5, 10 × 10, 15 × 15 and 20 × 20. The standard deviation of errors goes down as the synchronization iteration increases. This demonstrates the convergence of the proposed GNA algorithm. It can also be seen from the figure that the standard deviation of errors for bigger network size is larger than that for smaller network size. The reason is that the transmission range is limited. The averaging operation can only be taken within a small cluster. If the network size is larger, meaning that more sensor nodes are in the network, of course after the same rounds of synchronization, the standard deviation of errors is bigger. The convergence rate is slower if the network size increases. Therefore, more rounds of synchronization are needed for global synchronization if the network size becomes larger.

## 6. Conclusions

This paper has proposed a novel internal clock synchronization solution for wireless sensor networks. It is a fully distributed, network-wide synchronization algorithm. Compared with several recent popular synchronization algorithms, the proposed GNA algorithm considers the propagation delay. The two-way message exchange is adopted for the compensation of the propagation delay. Based on this new coming message exchange, a group average scheme was proposed. The random elected sensor node collects the offset and skew rate from neighbors and takes group averaging, which would achieve fast clock convergence. The analytical analysis of offset and skew compensation is described. The performance of the proposed GNA is fully simulated and analyzed for different kinds of offset and skew rate compensation scenarios. The simulation results showed that the proposed GNA algorithm achieves better performance in terms of accuracy and convergence speed than other algorithms like FWA and CWA. Future work would focus on full mathematical interpretation of clock synchronization in WSNs, energy consumption analysis and hardware testbed implementation.

## Figures and Tables

**Table 1.** Simulation parameters.

**Parameters**	**Values**	**Parameters**	**Values**
Network size	10 × 10	Initial offset	−1000 ∼ +1000
Synchronization rounds	10	Initial relative skew rate	<20 ppm
Initial sync time	2	Algorithms	FWA, CWA, GNA
Transmission range	1	Multiple runs	100
